# Prevalence and Risk Factors of Sexually Transmitted Infections and Cervical Neoplasia in Women from a Rural Area of Southern Mozambique

**DOI:** 10.1155/2010/609315

**Published:** 2010-07-11

**Authors:** Clara Menéndez, Xavier Castellsagué, Montse Renom, Jahit Sacarlal, Llorenç Quintó, Belen Lloveras, Joellen Klaustermeier, Janet R. Kornegay, Betuel Sigauque, F. Xavier Bosch, Pedro L. Alonso

**Affiliations:** ^1^Manhiça Health Research Center, Rua 12, Manhiça, CP 1929, Maputo, Mozambique; ^2^Barcelona Center for International Health Research (CRESIB), Hospital Clinic, Institut d'Investigacions Biomedicas August Pi i Sunyer (IDIBAPS), Universitat de Barcelona, Villarroel 170, 08036 Barcelona, Spain; ^3^Cancer Epidemiology Research Program, Institut Català d'Oncologia (ICO), Institut d'Investigació Biomèdica (IDIBELL), L'Hospitalet de Llobregat, 08907 Barcelona, Spain; ^4^Parc de Recerca Biomèdica de Barcelona (CIBER-ESP), Doctor Aiguader, 88, 8003 Barcelona, Spain; ^5^Roche Molecular Systems, Alameda, CA 94588, USA; ^6^National Directorate of Health & National Malaria Control Program, Ministry of Health, Mozambique; ^7^RTICC, Instituto de Salud Carlos III, C/Sinesio Delgado 4-6, 28029 Madrid, Spain

## Abstract

There is limited information on the prevalence of sexually transmitted infections and the prevalence of cervical neoplasia in rural sub-Saharan Africa. This study describes the prevalence and the etiology of STIs and the prevalence of cervical neoplasia among women in southern Mozambique. An age-stratified cross-sectional study was performed where 262 women aged 14 to 61 years were recruited at the antenatal clinic (59%), the family-planning clinic (7%), and from the community (34%). At least one active STI was diagnosed in 79% of women. Trichomonas vaginalis was present in 31% of all study participants. The prevalence of Neisseria gonorrhea and Chlamydia trachomatis were 14% and 8%, respectively, and Syphilis was diagnosed in 12% of women. HPV DNA was detected in 40% of women and cervical neoplasia was diagnosed in 12% of all women. Risk factors associated with the presence of some of the STIs were being divorced or widowed, having more than one sexual partner and having the partner living in another area. A higher prevalence was observed in the reproductive age group and some of the STIs were more frequently diagnosed in pregnant women. STI control programs are a priority to reduce the STIs burden, including HIV and cervical neoplasia.

## 1. Introduction

Sexually transmitted infections (STIs) remain a major public health problem in developing countries [1–5]. The HIV/AIDS pandemia [6] has increased the awareness about other STIs. However, their true scope and impact are still mostly unknown. Most of the burden of STIs is borne by women, yet women are less likely than men to seek treatment for STIs [7, 8]. In developing countries, complications related to STIs are a major cause of mother and child mortality and morbidity during pregnancy [9–11] as well as to the high incidences of cervical cancer [12, 13]. Previous data from Mozambique [14–16] shows alarmingly high incidences of cervical cancer and congenital syphilis which suggests that STIs prevalence is likely to be high. In the context of a steady increase of HIV prevalence [17] and in an era where access to health care and prevention systems are still weak in most parts of sub-Saharan Africa, further information to guide effective STIs prevention strategies as well as diagnostic and management policies is needed. 

Accordingly, a descriptive study on the prevalence and risk factors for STIs and cervical neoplasia among women living in rural Mozambique was carried out in order to contribute to generate further information.

## 2. Methods

### 2.1. Study Area and Population

The study took place in the Manhiça District, southern Mozambique, where the Centro de Investigação em Saúde de Manhiça (CISM) is located and approximately at 80 Km from the capital, Maputo.

The population in the Manhiça District lives in a semi-urban and rural setting and crop farming is the main activity in the area. Illiteracy is prevalent, being 24% among men and 47% among women. While 66% of men and 49% of women have had primary education, only 9% of men and 4% of women have had secondary education, and less than 1% of both men and women have gone beyond their secondary education [18]. An ongoing demographic surveillance system, that has been described in detail elsewhere is in place, covering a population of around 82,000 inhabitants, at the time of the study it covered a population of 36.000. Population data is collected on a semestral basis with a biannual census of the population [19].

The population structure is similar to that of many developing countries with 54% of the population being less than 20 years old.

The adjacent Manhiça District Hospital (MDH) is a 110-bed health facility with antenatal care clinic (ANC) services as well as a family planning clinic. 

At the time when the study was carried out, screening and treatment for syphilis were provided at the ANC. Residents are mostly subsistence farmers or employees in a nearby sugar cane processing factory. A high number of the adult male population of the area migrates to South Africa to work at the mines and many other move to the capital of Mozambique for job purposes as well.

### 2.2. Study Design

This is a cross-sectional, age-stratified study of STIs and cervical neoplasia in women. Two hundred and sixty two women aged 14–61 were recruited between August and October 2000. Fifty-nine percent were enrolled at the ANC and 7% at the family planning clinic (FPC) of the MDH. The remaining 34% were randomly selected from the community using the DSS census from the older than 50-year-old female population. Women recruited at the community were invited by the field workers to the hospital to complete the study visit. Attendance to the ANC is high and it is estimated that approximately 90% of pregnant women are visited at least once at the ANC. Women were offered an informed consent and no study procedures were performed before acceptance to the study was given by either signing or thumb printing the document. The refusal rate to participate in the study was 5%. The inclusion criteria were to attend the ANC or the FPC and for the community participants to be in the list randomly produced from the census. Exclusion criteria were exclusively their refusal to sign the informed consent. The study visit consisted of a gynecological exam, collection of cervical samples, and 5 mL of blood by venipuncture. During the visit, relevant information regarding the participant education, socioeconomic status (see Table 1), reproductive history and lifetime sexual behavior was collected as well. In accordance with the Ministry of Health (MOH) guidelines, unlinked HIV testing was done anonymously since, at the time of the study, there were no voluntary counseling and testing services, nor were antiretroviral drugs available. Women did not receive STIs presumptive treatment and they were offered free treatment according to national guidelines if diagnosed with an active treatable STI. 

Women with a Pap smear compatible with cervical intraepithelial dysplasia/neoplasia (CIN) or carcinoma were referred for histological confirmation and appropriate clinical management to the Maputo Central Hospital.

### 2.3. Laboratory Methods

The rapid plasma reagin (RPR, Syphacard, Wellcome, USA) test was used for syphilis diagnosis and results were confirmed using an enzyme immunoassay (CAPTIA Syphilis-G, Trinity Biotech, Dublin, Ireland). CT DNA was detected on cervical samples by the CT-ID assay (Digene Corporation, Silver Spring, MD, USA). Neisseria gonorrhea (NG) was identified by Gram stain and culture (Thayer-Martin, Bethesda, MD, USA) of the cervical mucosa. Trichomonas vaginalis was detected by direct microscopic examination of went-mount preparations of the vaginal discharge. Anti HSV-2 antibodies were determined using an ELISA kit from MRL Diagnostics (Cypress, CA, USA). Anti HIV antibodies were detected by Imx HIV-1/HIV-2 III Plus EIA (Abbot Diagnostics, IL, USA). Antibodies against Hepatitis B virus (anti-HBc) and HBsAg were detected using the ELISA technique ETI-AB-COREK-2 and ETI-MAK-3 (DiaSorin). HPV DNA was determined from a cervical swab by the reverse line-blot (RLB) strip-based detection system [20–22] and further improved by using the PGMY09-PGMY11 primer system. Cervical smears were processed and read following standard procedures. Antibiotic sensitivity was assessed by standard techniques using disc diffusion methods and interpreted according to the NCCLS criteria (National Committee for Clinical Laboratory Standards, 2000).

### 2.4. Statistical Methods and Definitions

Syphilis infection was defined as a RPR positive test confirmed by ELISA for *T. Pallidum* IgG antibodies. The variable “any treatable STI” was created for women with at least one of the following: syphilis, gonoccocal infection, *Trichomonas vaginalis* or CT. The variable “any active STI” included women with any of the following: syphilis, gonoccocal infection, *Trichomonas vaginalis*, CT, HPV or HIV infection. HSV was not included in this latter group because no confirmation of active disease laboratory analysis was performed during the study. Percentages were compared by the uncorrected *χ* ^2^ test and the Fisher's exact test. Odds ratio (OR), and 95% Confidence Intervals (CI) were estimated by logistic regression models. Adjustments for age and ethnic group were performed in the multivariate models. Age was categorized in tertiles (14–26, 27–42, and 43–61) for the analysis of risk factors to allow for small numbers or absence of cases in some of the outcomes. 

Multivariate models for STs and cervical neoplasia were estimated using a forward-stepwise procedure, with 0.05 from the Likelihood-ratio test as significance level for addition to the model. Potential independent variables for such models were the participant education, socioeconomic status, reproductive history and lifetime sexual behavior as well as other coinfections (STIs other than the one used as dependent variable for each model). Age and ethnic group were forced to be part of all multivariate models in order to adjust for them.

The confidence intervals for the proportion of STIs in Table 2 were calculated by the Exact (or Clopper-Pearson) method, based on the binomial distribution.

The analysis was performed using STATA software (Stata, East College Station, TX, USA).

## 3. Results

### 3.1. Demographic Characteristics

The median age at first sexual intercourse was 16, and 43% initiated sexual intercourse at age 15 years or younger (Table 1). About 11% of women had been married more than once and a third of their husbands had previously lived with 2 or more different women. The husbands were, on average, 5 years older than their wives (data not shown).

Women recruited at the FPC had a higher literacy level (47% versus 30% and 15% from the ANC and the community, resp.; *P* ≤ .001), and had more than one sexual partner in a higher percentage in comparison to the other groups (58% versus 41% and 33% from the ANC and the community, resp.; *P* = .047).

### 3.2. Prevalence of STIs and Cervical Neoplasia

Vaginal discharge was observed in 68% of cases, cervical mucous in 35%, anogenital warts in 5%. Overall prevalence of STIs is shown in Table 2. 

Nearly 70% (179/254) of all women and 75% (112/148) of pregnant women had at least one active STI. This prevalence reached 82% in the 14–20 years age group, and decreased significantly with age, dropping to 44% in the oldest group (51–61 years). Over half (51%) of the participants had at least one treatable STIs. We defined treatable taking into account what was available for treatment as per national policy guidelines at the time of the study.

The most common STI was Trichomonas vaginalis found in 31% of women. 

**Figure 1:**
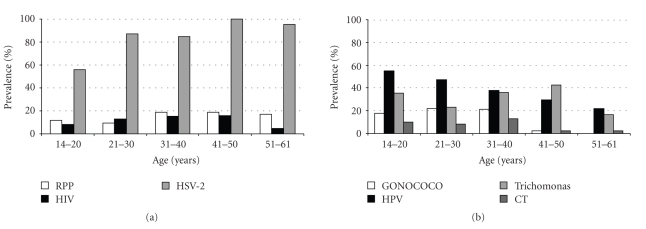
Seroprevalence of syphilis, HIV and HSV-2 (a) and prevalence of gonococcal, HPV, trichomonal and chlamydia trachomatis (CT) infections (b).

The overall prevalence of gonoccocal infection was 14% (34/250). It was 19% (27/145) in pregnant women and 33% (6/18) among women attending the FPC. Prevalence was associated with age (*P* < .001), being more prevalent in the younger age group. Antimicrobial sensitivity of the NG was 61% for erythromycin, 18% for penicillin and ampicillin, 15% for chloramphenicol, 9% for gentamicin and 3% for cotrimoxazol. Sensitivity to ceftriaxone, spectinomycin and azythromycin was not assessed. CT DNA was detected in 7.5% (19/253) of cervical samples. The prevalence was lower in the 41–61 age group, but there was no statistically significant association with age. Among pregnant women, the prevalence was 10% (15/151). Thirty-nine women had a RPR positive test (15%), of which 31 (75%) were confirmed by EIA, giving an overall syphilis prevalence of 12% (31/258). The prevalence increased with age, but this relationship was not statistically significant (*P* = .67). Among pregnant women, syphilis prevalence was 10% (15/149). Overall prevalence of IgG antibodies against *T pallidum* was 39% (100/257), increasing significantly with age (*P* < .001). Overall seroprevalence of HIV was 12% (30/256) and 21% (4/19) among women attending the FPC. The highest prevalence was found in the 31–50 age group (15%; 16/104), but there was no significant relationship with age. Anti-HSV-2 antibodies were found in 83% (213/257) of all participants. This frequency increased significantly with age (test for trend *P* < .001). The lowest prevalence (56%; 28/50) was in the youngest age group. HBsAg was detected in 8% (20/255) and anti-HBc in 62.5% (160/256) of women, respectively. The overall prevalence of HPV DNA was 40% (100/253), the highest was in the youngest age group (test for trend *P* < .001), and decreased with age (see Figure 1). HPV genotypes have been already published [23] Cervical neoplasia was detected in 30 women (12.2%): 5.3% (13/245) with LSIL (low-grade squamous intraepithelial lesions that include CIN I), 6.5% (16/245) with HSIL (high-grade squamous intraepithelial lesions that include CIN II and III), and one woman (0.4%) with carcinoma.

### 3.3. Sociodemographic, Behavioral Risk Factors and STIs Coinfection

Table 3 shows the multivariate analysis results for sociodemographic and sexual behavior-related risk factors for each STI and cervical neoplasia.

Reporting more than one lifetime sexual partner was the only factor statistically significant related to cervical gonoccocal infection. Observation of vaginal secretion or cervical mucous during clinical exam was not associated with gonoccocal infection. The number of lifetime sexual partners and the marital status were significantly associated with the diagnosis of syphilis, and the presence of HPV DNA was associated with a nearly three-fold higher risk (OR = 3.2; 95% CI 1.4, 7.5). Abnormal cervical cytology was associated with HPV DNA detection in cervical cells (OR = 10.1; 95% CI 3.7, 27.5). The presence of anogenital warts was also related to HPV infection (OR = 4.2; 95% CI 1.0, 17.8). A positive test for syphilis was associated with HPV DNA detection (OR = 4.1; 95% CI 1.9, 9.1). In the adjusted analysis, being divorced or widowed was significantly associated with HIV infection. HIV seropositivity prevalence rose with increasing severity of cervical abnormalities: 9% in women with normal cytology, 16% in women with ASCUS, 17% in women with LSIL, and 19% in women with HSIL, and the only woman with carcinoma (test for trend *P* = .02). HIV seropositive women were more likely to have HPV DNA detected in their cervices, but the differences were not statistically significant (47% versus 37%; OR = 1.6; 95% CI 0.5, 3.5). CT infection was not significantly associated with any social or behavioral risk factor. No relationship between detection of this infection and either vaginal secretion or cervical mucous was identified in the multivariate analysis. 

### 3.4. STIs Risk by HIV Status

Except for HBsAg, there was a consistent association trend for the presence of f all STIs in HIV positive women. However, only CT DNA detection and presence of cervical abnormalities reached borderline statistical significance (Table 4).

## 4. Discussion

Although none of the participants were selected based on their STIs seeking behavior, 70% and 50% of women presented with at least one active or treatable STI. It is generally assumed that STI prevalence is higher in urban residents [24]. However, these findings show that the burden of STIs in rural areas may be underestimated. The STI prevalences found were higher than those reported from other rural areas in sub-Saharan Africa [25, 26]. The high migration rate might partially explain the high frequency of STIs in these women [4].

Vaginal discharge showed no correlation with the presence of infection, which suggests that syndromic management alone is unlikely to have a major public health impact in controlling STIs and HIV transmission in women [27–29]. 

On the contrary, the presence of anogenital warts was significally correlated with the presence of any treatable STI. Most pregnant women had either an active (75.6%) or treatable (55%) STI. These frequencies were higher than what has been reported in the region [7, 30] and in a urban area of northern Mozambique (51%) [31]. The most frequent STI was Trichomonas vaginalis which was found in 31% of women, and taking into account that the direct examination only detects approximately a 60%–70% of infections it is reasonable to assume that we are underestimating its prevalence. The high prevalence of gonorrhea, CT, and syphilis are particularly alarming due to their potential impact on the newborn. At the time of the study, the only control program in place was that of syphilis prevention. These results call for different approaches to STIs prevention of the main STIs in both pregnant women and their partners. In Mozambique, current guidelines recommend ciprofloxacin in cases of vaginal discharge [32]. However, this drug is rarely available and kanamycin plus erythromycin is the combination still most frequently used. Sensitivity for kanamycin was not tested but it can be assumed to be similar to that of gentamicin (9%), which may partly explain the high frequency of gonoccocal infection observed. These are still the only available results on NG sensitivity in the country, and reflect the difficulties that countries with limited resources face in reconciling their health policies with some recommendations. 

CT infection prevalence was 8% overall and the 14–20 years old group had a higher prevalence of CT infection (10%) than that reported from rural Tanzania (2.4% of female adolescents) [33]. This discrepancy may be explained by the recruitment sources of our study being more likely for our participants to have had more sexual partners. These data are consistent with the evidence that girls and young women are more susceptible to CT infection than older women, with the consequent risk, among others, of infertility [34].

Syphilis prevalence between 1.6% and 9.8% in rural areas [16] to 18.3% in Maputo has been reported among women in Mozambique [11]. In this study, syphilis was detected in 12% of all women and in 10% in the youngest age group. These figures are also higher than those from rural areas of South Africa (8%) [8] and Tanzania (9.1% of all women, 6.6% of adolescents) [35]. Again, the different selection of individuals may explain the different rates found in the current study. However, syphilis prevalence was also high in the oldest age groups, where most women were recruited from the community and therefore not exposed to the potential selection bias.

The overall prevalence of HSV-2 antibodies was high (83%). Among 14–20 year old women the prevalence was 56%, higher than that reported in another study from rural Africa (27%) [36]. HSV-2 is of much interest in Africa because it increases the risk of HIV transmission [37]. Although the information on HSV-2 is based on seroprevalence of antibodies, HSV-2 seropositivity has been proposed as a marker of sexual risk behavior among adolescents [36]. These observations support the latter, and suggest that there is high-risk sexual behavior at very young ages. 

The overall prevalence of HPV infection was high, especially in the youngest age group where over 50% were infected. HIV and co infection with other STIs are highly prevalent in this population, and this is known to increase the risk of progression from cervical HPV infection to cervical neoplasia and invasive cancer [38]. The HIV seroprevalence (12%) found was still lower than that reported from other areas of Southern Africa [39]. However, it could be foreseen that with this high STIs burden, the epidemic could rise dramatically if no effective STIs prevention is implemented. HIV prevalence estimates among ANC attendees in this area have reached 24% confirming this suspicion [40]. The risk factors for STI detection identified are consistent with those observed in other studies [41]. However, the indirect information obtained through questionnaires makes the data on behavioral risk factors subject to bias and difficult to reproduce [42, 43]. The main limitation of this study is that many women were not selected from the community and that the size and age distribution of the three recruitment sources are not comparable. These may limit the extrapolation of these findings to the general population. However, the similar rate of syphilis found among the community-based recruited women suggests that the potential selection bias may not be relevant. 

Our data is still to date the only available information on the prevalence of STIs and cervical cancer among women in Mozambique. A study performed in 2004 in an urban area of Mozambique reported a remarkable decline in STIs, which was attributed to successful implementation of STIs prevention strategies in that area [31].

We have to mention that one of the limitations of the study is that it may be underpowered for some of the tested associations.

In conclusion, the burden of disease associated with STIs borne by African women in rural areas, and the implications for the acquisition and transmission of HIV and cervical neoplasia, are enormous. Reproductive health programs do not easily reach adolescents and older women, so specific approaches to target them should be envisioned and implemented. In pregnant women, there is a need for a more aggressive approach to avoid the harmful effects associated with STIs on mothers and children. For HPV infections and cervical cancer, recent development of vaccines [44] offers great hope, but the challenge is that they become affordable for developing countries. There is an urgent need to develop interventions in order to avoid the burden of morbidity due to STIs and the further spread of HIV in rural African populations.

##  Funding

The financial support was provided by the Spanish Fondo de Investigación Sanitaria (FIS01/1236). The “Centro de Investigação em Saúde da Manhiça” (CISM), receives major core funding from the Spanish Agency for International Cooperation.

##  Conflict of Interest

None declared.

##  Ethical Approval 

Ethical clearance was provided by the Mozambican Ministry of Health, the Ciutat Sanitaria i Universitaria de Bellvitge and the Hospital Clinic Ethics Committees, Barcelona.

##  Author's Contributions

All the authors contributed to the design of the paper. Jahit Sacarlal, Betuel Sigauque, Montse Renom, and Clara Menéndez carried out the clinical work. Llorenç Quinto led the statistical analysis. Belen Lloveras, Joellen Klaustermeier, and Janet R Kornegay were responsible of the laboratory tests. Xavier Castellsague, Xavier Bosch, and Pedro Alonso contributed to the overall supervision of the paper. All authors contributed to the writing up of the paper that was led by Clara Menendez and Xavier Castellsague.

## Figures and Tables

**Table 1:** Characteristics of the study women.

		*n*	%
Age group	14–20	51	19
	21–30	62	24
	31–40	54	21
	41–50	53	20
	51–61	42	16

Ethnic group	Shangana	208	79
	Other	54	21

Education	Reads and/or writes	81	31
	Neither reads nor writes	181	69

Marital status	Never married	20	8
	Currently married	212	81
	Divorced or widowed	30	11

Number of women living with husband	1	185	71
	>1	72	27
	Unknown	5	2

Husband's job	Farmer	59	23
	Driver	8	3
	Miner	51	19
	Manual worker	44	17
	Shop keeper	7	3
	Civil servant	10	4
	Other	45	17
	Unknown	38	15

Husband's place of residence	Manhiça	133	51
	Maputo	27	10
	South Africa	52	20
	Unknown	50	19

Parity	None	46	18
	1	29	11
	2 to 5	99	38
	>5	85	32
	Unknown	3	1

Pregnant now	No	111	42
	Yes	151	58

Age at first sexual intercourse	12–15	52	20
	16-17	43	16
	18–20	26	10
	Unknown	141	54

Number of sexual partners in lifetime	Never had	3	1
	1	149	57
	>1	103	39
	Unknown	7	3

**Table 2:** Prevalence of STIs and cervical neoplasia in women from Manhiça, Mozambique.

	*n*/total	Prevalence (%)	95% Confidence interval
Gonococcal infection	34/250	14	(10; 19)
Syphilis (RPR & IgG)	31/258	12	(8; 17)
HPV DNA	100/253	40	(33; 46)
HIV antibodies	30/256	12	(8; 16)
HSV-2 antibodies	213/257	83	(78; 87)
Chlamydia trachomatis	19/253	8	(5; 11)
Trichomona vaginalis	78/254	31	(25; 37)
HBsAg antibodies	20/255	8	(5; 12)
Anti-HBc antibodies	160/256	63	(56; 68)
Any treatable STI*	128/252	51	(44; 57)
Any active STI**	179/254	70	(64; 76)
Cervical neoplasia	30/245	12	(8; 17)

*Any treatable STI: syphilis (RPR confirmed by IgG), gonococcal infection, trichomona vaginalis or chlamydia trachomatis.

**Any active STI: syphilis (RPR confirmed by IgG), gonococcal infection, trichomona vaginalis, chlamydia trachomatis, HPV or HIV infection.

**Table 3:** Significant variables according to the multivariate analysis of risk factors for STIs and cervical neoplasia.

Infection	Variable		Multivariate OR*	(95% CI)	*P*-value**
Gonococcal infection	Number of sexual partners	1	1		.014
		>1	2.8	(1.2; 6.2)	

Syphilis (RPR & IgG)	HPV	No	1		.005
		Yes	3.2	(1.4; 7.5)	

Syphilis (RPR & IgG)	Marital status	Never married	1		.005
		Married now	0.7	(0.1; 3.9)	
		Divorced or widowed	3.6	(0.6; 21.9)	

HPV	Cervical neoplasia	No	1		<.0001
		Yes	9.8	(3.7; 26.3)	
		Unknown	2.7	(0.6; 12.0)	

HPV	RPR	No	1		<.0001
		Yes	4.1	(1.9; 9.1)	

HPV	Anogenital warts	No	1		.054
		Yes	4.2	(1.0; 17.8)	

HIV	Marital status	Never married	1		.005
		Married now	0.5	(0.1; 2.3)	
		Divorced or widowed	2.7	(0.5; 16.0)	

HIV	Parity	None	1		.02
		1 child	0.7	(0.1; 3.6)	
		2 to 5	0.6	(0.1; 3.0)	
		more than 5	4.3	(0.7; 26.9)	

HSV-2	Age at first child	Never had	1		.014
		13–18	3.7	(1.1; 12.9)	
		19	48.7	(3.0; 792.7)	
		20–28	27.3	(3.1; 241.2)	
		Unknown	2.3	(0.5; 9.7)	

HSV-2	Selection of women	Antenatal clinic	1		.006
		Family planning	0.1	(0.0; 0.8)	
		Other	0	(0.0; 0.3)	

Trichomona vaginalis	Husband's place of residence	Manhiça	1		.002
		Maputo	2.5	(1.0; 6.3)	
		South Africa	1.2	(0.6; 2.4)	
		Unknown	0.2	(0.1; 0.6)	

Trichomona vaginalis	Anogenital warts	No	1		.018
		Yes	7.1	(1.4; 36.5)	

HBsAg	Number of women living with husband	1	1		.028
		>1	0.1	(0.0; 0.8)	

Any treatable STI^#^	Anogenital warts	No	1		.007
		Yes	11	(1.9; 63.1)	

Any active STI^##^	HSV-2 antibodies	No	1		.0029
		Yes	2.4	(1.1; 5.4)	

Any active STI^##^	Chlamydia trachomatis	No	1		.042
		Yes	2.3	(1.0; 5.1)	

Cervical neoplasia	HPV	No	1		<.0001
		Yes	10.1	(3.7; 27.5)	

Cervical neoplasia	RPR	No	1		.068
		Yes	0.2	(0.1; 1.1)	

*Adjusted by age and ethnic group.

**Likelihood Ratio Test versus model with variable removed.

^#^Any treatable STI: syphilis (RPR confirmed by IgG), gonococcal infection, trichomona vaginalis or Chlamydia trachomatis.

^##^Any active STI: syphilis (RPR confirmed by IgG), gonococcal infection, trichomona vaginalis, Chlamydia trachomatis, HPV or HIV infection.

**Table 4:** Prevalence and risk of STIs and cervical neoplasia by HIV status.

STD by HIV	HIV (−)		HIV (+)		Adjusted* OR	95% Cl	*P*
	*N* = 226		*N* = 30				
	*n*	%	*n*	%			
Gonococcal infection	27	12	6	20	1.9	(0.7; 5.5)	.219
Syphilis (RPR & IgG)	26	12	5	17	1.6	(0.5; 4.5)	.408
HPV DNA	83	37	14	47	1.6	(0.7; 3.5)	.262
HSV-2 antibodies	185	82	27	90	2.1	(0.6; 7.6)	.259
Chlamydia trachomatis DNA	14	6	5	17	3.1	(1.0; 9.6)	.051
Trichomona vaginalis	67	30	9	30	1.0	(0.4; 2.4)	.966
HBsAg anribodies	19	8	1	3	0.4	(0.1; 3.0)	.359
Anti-HBc antibodies	137	61	22	73	1.8	(0.8; 4.3)	.172
Any treatable STI**	107	47	18	60	1.7	(0.8; 3.8)	.190
Cervical neoplasia	23	10	6	20	2.5	(0.9; 7.0)	.088

*Adjusted by age and ethnic group.

**Any treatable STI: syphilis (RPR confirmed by IgG), gonococcal infection, trichomona vaginalis or chlamydia trachomatis.
